# Double blinding requirement for validity claims in cognitive-behavioral therapy intervention trials for major depressive disorder. Analysis of Hollon S, *et al*., Effect of cognitive therapy with antidepressant medications vs antidepressants alone on the rate of recovery in major depressive disorder: a randomized clinical trial

**DOI:** 10.12688/f1000research.6954.1

**Published:** 2015-08-27

**Authors:** Douglas Berger

**Affiliations:** 1 Meguro Counseling Center, Tokyo, Japan

**Keywords:** psychotherapy, cognitive-behavioral therapy (CBT), outcome studies, blinding, clinical trials

## Abstract

This paper will focus on problems in the inability to double-blind cognitive-behavioral therapy (CBT) studies for major depressive disorder (MDD), and provides an analysis of a recently published study to show how this problem can lead to faulty conclusions.

A study by Hollon *et al.* published in JAMA Psychiatry that compared an antidepressant medication-only arm with a combined CBT/antidepressant arm concluded that the cognitive therapy/antidepressant combination enhanced the recovery rates compared with antidepressant alone, and that the magnitude of this increment nearly doubled for patients with more severe depression.

We propose that for subjects with greater severity, there could have been both antidepressant efficacy as well as more hope and expectation in the group who knew they had received combined cognitive therapy/medication, leading to an erroneous conclusion of greater efficacy for the combined group. The large subject number in this study could easily lead to an erroneous finding on statistical testing as a small amount of bias in the subjects adds-up.

We opine that the conclusions of unblind CBT outcome research in conditions with subjective endpoints such as MDD need to be given with great caution. The validity of CBT (and its derivatives such as dialectical behavioral therapy) for indications other than MDD is also part of a larger problem in  the inability to blind outcome studies for these interventions.

The validity of cognitive-behavioral therapy (CBT) efficacy for major depressive disorder (MDD) is widely accepted and is based largely on clinical intervention studies of CBT in MDD. However, clinical trials for CBT cannot be carried out under double-blind conditions as would be required of pharmacotherapy (or other somatic therapies), thus the rigor of CBT interventional studies is quite different from those modalities that can be studied under double-blinded conditions ^1, 2^.

Treatment allocation cannot be blinded in CBT studies because the subjects have to actively participate in cognitive restructuring tasks. More than just saying a study was “blinded”, absolute concealment of what treatment was allocated is crucial in order to avoid bias ^3^.

CBT trials are sometimes stated to be, “single-blind” because the persons who rate the symptoms that subjects report are blind to the treatment allocation of the subject. The term “single-blind”, however, should be used with caution as single-blind is defined as the condition when subjects are blind, not the raters ^4^. Blind (or “masked”) raters only record whatever bias may be in the subjective reports of the subjects that can be swayed by the unblinded conditions. Emphasizing that raters are blind in a CBT study can distract from the issue that subjects and treaters are not blind.

Allocation concealment is crucial for indications with subjective outcomes as in MDD ^3^. During a clinical trial, subjects with MDD report changes in the severity of subjective depressive symptoms that may be influenced by an expectation or hope for improvement ^3^. Only interventional studies for indications with objective endpoints can ignore potential bias from lack of blinding. For example, mortality rates, MI incidence, stroke, etc. where random error is small ^1^. In this line, a meta analysis of CBT trials that controlled for blinding found treatment effects to be small in MDD ^5^.

However, studies continue to report positive results of unblinded trials without voicing strong caution on the validity of the results. Hollon *et al.* in the October 2014 issue of JAMA Psychiatry compared an antidepressant medication only arm with a combined cognitive therapy/antidepressant arm ^6^. All the subjects who received antidepressants did so under unblinded conditions. The cognitive therapy subjects and their treaters were also unblind to the treatment given. The study concluded that the cognitive therapy/antidepressant combination enhanced the rate of recovery compared with antidepressant alone, and that the magnitude of this increment nearly doubled for patients with more severe depression with little evidence of benefit for patients with less severe MDD. Only one line at the end of the discussion noted that the unblinded conditions could be a limitation.

An alternative conclusion could just as easily be that patients with greater severity MDD may have included more patients with a medication-responsive depression ^7^. For those subjects with greater severity, there could have been both antidepressant efficacy as well as more hope and expectation in the group who knew they had received combined cognitive therapy/medication leading to an erroneous conclusion of greater efficacy for the combined group. A large sample size (N) as in this study is not necessarily a sign of robust results. A large N can create a significant finding on statistical testing as a small amount of bias in the subjects adds-up ^1^. Our alternative conclusion may also be incorrect, the important issue is that the lack of allocation concealment in the study design does not allow any valid conclusion to be made either way. The antidepressant in each arm of the study provides the same amount of hope and expectation; the CBT arm has the added potential for bias from hope and expectation.

In addition, combining and comparing antidepressants that have market approval based on double-blinded placebo controlled outcome research with CBT, heretofore never studied under double-, or single-blinded conditions, in the same unblinded study is a serious problem. Handicapping one intervention group (antidepressants without the double-blinded placebo control needed for proof of efficacy), while providing advantage to another intervention group (unblinded CBT with no psychotherapy placebo which allows bias in one arm) which is then mixed with the handicapped group, confounds the study conditions and invalidates the design logic of a clinical trial.

To be sure, interventional studies for somatic therapies such as medications may also have elements of allocation non-concealment requiring caution in their interpretation. While medications can feasibly be blinded, side-effects may expose a subject to the fact that they are in the active-drug arm of a study. An exit analysis on the proportion of subjects in a study that correctly guessed the treatment arm they were in should be done, and the results of any study in an indication with subjective endpoints such as MDD that has evidence of unblinding should be suspect to have bias. Psychotherapy treatment, on the other hand, is virtually impossible to hide from the subject who is openly given the treatment. Whether medication, psychotherapy, or other intervention, no valid scientific assessment of efficacy can be made if a hurdle such as double-blinding in the study design of an indication with subjective endpoints is not rigorously implemented.

Authors must state clearly when an intervention cannot be studied with rigor, and conclusions need to be given with great caution when studies with subjective endpoints are unblinded. There is no regulatory authority like the FDA to review and approve a psychotherapeutic intervention for MDD, so that both professionals and society at large alike are dependent on the sound-bite conclusions made by authors and commentators on the results reported.

The critical problem of the inability to double-blind CBT clinical trials for MDD requires further evaluation by research groups who do not have a vested interest in CBT or related therapies. The validity of CBT (and its derivatives such as dialectical behavioral therapy) for indications other than MDD is part of a larger problem in the inability to blind outcome for these interventions.
