# Supplementary material for: Double blinding requirement for validity claims in cognitive-behavioral therapy intervention trials for major depressive disorder. Analysis of Hollon S,  et al., Effect of cognitive therapy with antidepressant medications vs antidepressants alone on the rate of recovery in major depressive disorder: a randomized clinical trial
Source: F1000Res. 2015 Aug 27;4:639. [Version 1] doi: 10.12688/f1000research.6954.1 (PMC4863672; doi:10.12688/f1000research.6954.1)
Supplement: Peer review history

# Peer review: Referee response for version 1

**DOI:** 10.5256/f1000research.7489.r13511

**Published:** 2016-05-09

**Authors:** Arif Khan

**Affiliations:** 1 Northwest Clinical Research Center, Bellevue, WA, USA

Dr. Berger has taken to task a sacred cow in our field.  Psychotherapy works and any challenges to it amount to sacrilegious position!  Unfortunately for the dogmatic, Dr. Berger is right.  It is not possible to truly 'blind' a psychotherapy study.  

Simply put, the model of blinded raters is limited as both the patient and therapist are not blinded.  Whether one likes it or not, the enthusiasm and conviction of a therapist is more than porous.  The Freudian model is that a cigar smoking therapist begets a cigar smoking patient.  

In the same note the sham therapist's conviction and enthusiasm (or a lack of it) will also be picked up by the patients.  We have shown that the level of blinding does change expectations and expectations for the psychotherapy + pharmacotherapy results in the expected increased benefit. ^1^

Having said this, it would be foolish of me to suggest that psychotherapy has no value.  After all, practice of medicine, psychiatry and psychology is intrinsically and intricately based on human interactions.  So, training and practicing of psychotherapy is essential, just like saying you have to know anatomy before you start surgery.  It is an essential skill in humanistic sciences.  However, just like proving you need to know anatomy before you cut somebody up surgically cannot really be tested using current models and the same applies to psychotherapy.  

In this context, it is important to note that different types of psychotherapy are better than other ones.  This is like testing a surgical knife for different surgeries.  All the psychotherapies (if practiced well) are effective just like all knives are effective.  

It is only a poor clinician who tries to treat a patient without the understanding and application of psychotherapy principles to distressed patients.  Here, I would like to note that Dr. Berger is a known psychotherapist.  Thus, his position is courageous and to be lauded.

I have read this submission. I believe that I have an appropriate level of expertise to confirm that it is of an acceptable scientific standard.

## References

[1] KhanAFaucettJLichtenbergPKirschIBrownWA: A systematic review of comparative efficacy of treatments and controls for depression. *PLoS One*.2012;7(7) : 10.1371/journal.pone.0041778 e41778 10.1371/journal.pone.0041778 22860015PMC3408478

# Peer review: Referee response for version 1

**DOI:** 10.5256/f1000research.7489.r10880

**Published:** 2015-10-26

**Authors:** Rebecca Graham

**Affiliations:** 1 Black Dog Institute, Prince of Wales Hospital, Randwick, NSW, Australia

Overall, a reasonably well-written summary of the complexities evident in CBT intervention trials for MDD. The only suggestion I would make is that the grammar should be checked carefully prior to publication - especially the correct use of commas. Other than that - would agree with indexation.

I have read this submission. I believe that I have an appropriate level of expertise to confirm that it is of an acceptable scientific standard.
